# Supplementary material for: Agreement between two large pan-cancer CRISPR-Cas9 gene dependency data sets
Source: Nat Commun. 2019 Dec 20;10:5817. doi: 10.1038/s41467-019-13805-y (PMC6925302; doi:10.1038/s41467-019-13805-y)
Supplement: Supplementary file 3 — Description of Additional Supplementary Files [file 41467_2019_13805_MOESM3_ESM.pdf]

Description of Additional Supplementary Files

File Name: Supplementary Data 1
Description: Experimental pipelines' specifications

File Name: Supplementary Data 2
Description: Cell lines screened in both studies

File Name: Supplementary Data 3
Description: Common dependencies in each study

File Name: Supplementary Data 4
Description: Gene NormLRT scores in both studies

File Name: Supplementary Data 5
Description: (a) Studies’ agreement across tissues; (b) Cell line molecular features used in the systematic inference of gene essentiality markers

File Name: Supplementary Data 6
Description: Dependency markers test results from the two individual studies

File Name: Supplementary Data 7
Description: Marker consistency via ROC analysis

File Name: Supplementary Data 8
Description: GO:terms enriched in the Broad-exclusive dependencies
